# The Combined Use of Metformin and Methotrexate in Psoriasis Patients with Metabolic Syndrome

**DOI:** 10.1155/2022/9838867

**Published:** 2022-04-22

**Authors:** Huynh Thi Xuan Tam, Luong Nguyen Dac Thuy, Ngo Minh Vinh, Tran Ngoc Anh, Bui Thi Van

**Affiliations:** ^1^Pham Ngoc Thach University of Medicine, Ho Chi Minh, Vietnam; ^2^University of Medicine and Pharmacy, Ho Chi Minh, Vietnam; ^3^Scientific Institute of Clinical Medicine and Pharmacy 108, Hanoi, Vietnam

## Abstract

**Objective:**

To evaluate the efficacy and safety of the combination of metformin and methotrexate (MTX) versus MTX monotherapy in treating psoriasis in patients with metabolic syndrome.

**Materials and Methods:**

A prospective clinical trial was conducted using metformin and MTX to treat psoriasis patients with metabolic syndrome. A treatment group of 35 psoriasis patients with metabolic syndrome was treated with MTX and metformin. A control group of 31 psoriasis patients with metabolic syndrome was treated with MTX only.

**Results:**

Patients treated with the combined regimen showed measured improvement in disease status compared to those treated with MTX monotherapy. The Psoriasis Area and Severity Index (PASI) scores of psoriasis patients with metabolic syndrome using the metformin and MTX combination were significantly lower than those treated with MTX only (*p* < 0.05). The combination treatment group also showed a significant decrease in blood sugar and triglyceride levels after 3 months (*p* < 0.05). However, there were no significant differences in subclinical indexes between the treatment and control groups.

**Conclusion:**

In this treatment sample, a combination of metformin and MTX in psoriasis patients with metabolic syndrome showed positive responses and no serious side effects.

## 1. Introduction

Psoriasis is a skin condition manifested by epidermal proliferation, abnormal differentiation of the stratum corneum, and capillary proliferation. Morphology of the disease includes patches, drops, generalized erythema, pustules, or nail damage [1]. Recent literature indicates that psoriasis is associated with increased morbidity and mortality from cardiovascular disorders, metabolic syndrome, and dyslipidemia, especially in severe and prolonged psoriasis cases [2–4]. These studies also demonstrate that the prevalence of metabolic syndrome in psoriasis patients is higher than in patients with other skin diseases. The pathogenesis of metabolic syndrome in patients with psoriasis is believed to have a connection with an increase in adipocytokines such as tumor necrosis factor-*α* (TNF-*α*) and adiponectin [2–4]. A recent study demonstrated that metformin—widely used to lower blood glucose concentrations in the treatment of diabetes patients—may be useful in combination with methotrexate (MTX) for the treatment of psoriasis [5]. Biochemical indicators suggest that both drugs have the same target: AMP-activated protein kinase (AMPK) [5]. MTX inhibits cellular DNA synthesis and is considered the “gold standard” in the treatment of psoriasis [6]. However, used in high doses over a long period of time, MTX has been shown to cause harmful side effects to the liver (hepatotoxicity), blood, bones, and lungs [7]. Studies in laboratory animals have demonstrated that the anti-inflammatory and antiproliferative effects of metformin may reduce the hepatotoxicity of MTX [8–11]. No clinical studies evaluating the clinical effectiveness of this drug combination in psoriasis patients have yet been published. Therefore, this study was conducted to evaluate the effectiveness and safety of combination therapy using metformin and MTX in the treatment of psoriasis patients with metabolic syndrome.

## 2. Materials and Methods

### 2.1. Materials

This single-blind clinical trial was conducted from June 2016 to October 2018 with 66 patients diagnosed with plaque psoriasis who were being treated at Ho Chi Minh City (HCMC) Hospital of Dermato-Venereology. All patients met full criteria to participate in this research.

This study was approved by the Board of Ethics at Pham Ngoc Thach University of Medicine. The author obtained informed consent from participants. The procedures followed were in accordance with the ethical standards of the responsible institutional committee on human experimentation and with the Helsinki Declaration of 1975, as revised in 2013.

#### 2.1.1. Diagnostic Criteria

*Psoriasis diagnostic criteria:* Psoriasis diagnosis is based on clinical features. Lesions are erythematous plaques with scales on the surface, and suggestive characteristics are a circumscribed border, noninfiltration, sites of predilection, mild or severe pruritus, and silvery scales.

*Metabolic syndrome diagnostic criteria:* According to the criteria of the National Cholesterol Education Program Adult Treatment Panel III (NCEP/ATP III) and the South Asian Modified (SAM)-NCEP, the diagnosis of metabolic syndrome is established when 3 of 5 factors are present [12] (Table 1).

#### 2.1.2. Inclusion Criteria

- Patients with both psoriasis vulgaris and metabolic syndrome between the ages of 18 and 70 years.
- Patients are nonalcoholic; liver and kidney function tests are normal.
- Patients consent to participate in this research study.

#### 2.1.3. Exclusion Criteria

- Patient is pregnant or lactating.
- Patient has been using systemic drugs to treat psoriasis—such as cyclosporine, retinoid, or immunologic therapy—for one month or more.
- Patient has acute or chronic infection.
- Contraindication to use metformin and methotrexate.

### 2.2. Methods

Study design*:* prospective, randomized placebo control study with convenient sampling. Psoriasis patients with metabolic syndrome were divided into two groups:

- Treatment group: 35 psoriasis vulgaris patients with metabolic syndrome were treated using metformin + MTX. MTX: started with 7.5 mg/week, divided into 3 doses q12 hr, and sustained for three months (12 weeks). Metformin: 500 mg/day after one meal.
- Control group: 31 psoriasis vulgaris patients with metabolic syndrome were treated using MTX only with the same dosage and usage.

#### 2.2.1. Result Evaluation

Both groups were evaluated using a number of factors.

- Treatment results were evaluated using the Psoriasis Area and Severity Index (PASI) before treatment and after treatment at 1 month, 2 months, and 3 months.
- Blood samples were taken before treatment and 3 months after treatment to test full blood count, urea, creatinine, aspartate aminotransferase (AST), alanine aminotransferase (ALT), *γ*-glutamyl transferase (GGT), cholesterol, triglycerides, and fasting glucose. The indexes of AST, ALT, GGT, triglycerides, total cholesterol, and HDL-C were measured using a HumaStar 600 machine. All tests were conducted in the laboratory of the HCMC Hospital of Dermato-Venereology.
- Clinical effects were measured by the percent of PASI reduction, according to the equation

(1) $$\begin{array}{c} \%\text{ PASI reduction}=\frac{\text{PASI before treatment}-\text{PASI after treatment}}{\text{PASI before treatment}\times 100}\times 100. \end{array}$$

Based on PASI reduction, there are 5 levels:

- Very good: PASI reduction 90–100%
- Good: PASI reduction 75%– < 90%
- Moderate: PASI reduction 50– < 75%
- Medium: PASI reduction 25– < 50%
- Bad or no effectiveness: PASI reduction <25%.

#### 2.2.2. Data Analysis

Data were analyzed using Stata 12 software. The data were analyzed using frequency, percentage, mean, standard deviation, and median. *χ*2 is used to identify the relationship between qualitative variables. The generalized estimating equation (GEE) methodology is used to analyze correlated data that otherwise could be modeled as a generalized linear model. For quantitative variables with normal distribution, we used the Student *t*-test to compare two mean values and analysis of variance (ANOVA) to compare more mean values. For quantitative variables with abnormal distribution, we used the Wilcoxon two-sample test.

## 3. Results

Research group characteristics are shown in Table 2. Treatment results of the study group are shown in Table 3. Treatment outcome of the control group is shown in Table 4. Results of treatment in two groups according to the rate of PASI reduction after 3 months are shown in Table 5. AST, ALT, and GGT before and after treatment of the two groups are shown in Table 6. Comparison of treatment results of two groups according to the venous blood glucose level is shown in Table 7. Comparison of treatment results of two groups according to the triglycerides index is shown in Table 8. Comparison of treatment results of two groups according to HDL cholesterol index is shown in Table 9. Comparison of treatments results of two groups according to total cholesterol is shown in Table 10.

## 4. Discussion

As shown in Table 2, no significant difference in age, disease duration, or PASI between the two groups was noted. The results of this combination therapy in Table 3 show that the mean PASI decreased each month, and more than 30% of the patients achieved PASI 75 (Table 5). Primarily, patients with “very good” PASI improvement accounted for 28.6%, which is similar to the results in the study by Singh and Bhansali [13]. According to the study by Singh and Bhansali, 85.7% of the patients achieved PASI 75 after treatment with metformin alone and the PASI index after 12 weeks reduced by 3.9%. However, in the group of patients treated with MTX alone, although the mean PASI underwent a decrease of 50.22% after three months (Table 4), there were no patients with PASI reaching “good” or “very good” levels, and this difference was statistically significant (*p* < 0.001) (Table 5). Saurat et al. found that after 16 weeks of treatment with increased dosage of MTX, only 36% of the patients achieved PASI 75 [14]. Reich et al. used MTX at a dose of 5–25 mg/week to treat moderate and severe psoriasis continuously for 24 weeks, yet only 27.6% of the patients completed the trial, while the remaining had to stop the treatment due to drug side effects. Worse in this study, only 39.9% of the patients achieved PASI 75 [15]. Both the studies by Saurat et al. and Reich et al. recorded a much lower percentage of patients achieving PASI 75 than our study. Thus, the addition of metformin in psoriasis patients with metabolic syndrome using MTX appears to contribute to improving patients' psoriasis. This result is also consistent with that in the study by El-Gharabawy et al. They concluded that MTX combined with metformin, a drug capable of modulating immune responses, could reduce the severity of psoriasis in early stages [16]. A case-control study with 36,702 patients also found that long-term adoption of metformin reduced psoriasis incidence in patients with diabetes [17].

Metformin can be incorporated with MTX in the treatment of psoriasis arthritis. A recent study showed that this combination is more effective in the treatment of psoriatic arthritis than MTX alone due to its higher anti-inflammatory effect [18]. Biochemical data suggest that both drugs act on AMPK [5]. This enzyme has a vital role in regulating metabolism and controlling many target tissues, such as the growth and functioning of T lymphocytes. Through the extracellular signal-regulated kinases 1/2 (ERK1/2) pathway, the cell cycle stops and thus inhibits cell growth, which is characteristic of psoriasis [10]. AMPK activation not only deactivates inducible nitric oxide synthase (iNOS), dendritic cells, T-cells, and monocytes/macrophages but it also stimulates interleukin 10 (IL-10) and transforming growth factor *β* (TGF-*β*), leading to anti-inflammatory effects [5]. El-Gharabawy et al. reported that combination metformin (850 mg) with topical therapy of psoriasis considerably lowered the number of T-CD4+ lymphocytes and the levels of IL-2, C-reactive protein (CRP), ceruloplasmin, ALT, and AST compared to untreated psoriasis patients [16]. Furthermore, numerous studies show that treatment with metformin reduced inflammatory markers and other cytokines, including TNF-*α* and interferon-*γ* [19, 20]. In keratinocytes, metformin has the ability to inhibit their growth through the mitochondrial-activated protein kinase pathway [10]. Moreover, metformin also constrains growth and proinflammatory cytokines via the rapamycin signal pathway in cultured human skin cells [20]. It is the growth-suppressing and anti-inflammatory properties of metformin that may be effective in psoriasis.

The results of the study did not show any serious treatment side effects in either group. Liver function, reported in Table 6, showed no significant change in AST and ALT indexes in either group. Only the GGT index in the MTX group increased significantly after 12 weeks. This result is inconsistent with that in the study by Laura et al., in which 188 patients were treated with MTX at a dose of 15 mg per week, and after 3 months, 10.6% of them had liver abnormalities and 2.1% needed to discontinue treatment with MTX [21]. According to a study by Kragaelle et al., the rate of increased ALT enzyme was 13% in the first year [22]. One reason for this discrepancy could be the difference in time and dosage. The present study only used a dosage of 7.5 mg/week of MTX over a period of three months. Still, according to the literature, liver toxicity is a concern with the long-term use of MTX. This may be caused by stress oxidation: an increase in reactive oxygen and nitrogen radicals, coupled with a decrease in antioxidant mechanisms, causes damage to hepatocytes [11].

Although there was no statistically significant difference, the GGT index of the experiment group of patients decreased after 12 weeks. In an animal clinical trial, the combination of metformin and MTX significantly reduced liver enzymes and bilirubin levels, shortened the prothrombin time, and significantly reduced thrombospondin-1 concentration [11]. Additionally, histopathology of liver tissue of the group treated with metformin demonstrated a significant improvement in the liver structure. Although the necrotic lesions remained, the severity was significantly lessened. We can speculate that metformin has a protective mechanism against MTX-induced hepatotoxicity. A study by Risk et al. revealed a defensive action of metformin against renal toxicity from MTX chemotherapy [23]. The antioxidant, antiapoptotic, and anti-inflammatory properties of metformin may serve as contributing factors to this hepatorenal protection.

Venous blood glucose levels in our experiment reduced significantly in the experiment group, which may be explained by the hypoglycemic ability of metformin. However, when comparing the two groups, there was not a significant difference (Table 7). The same pattern was observed for triglycerides, HDL, and cholesterol indexes (Tables 8–10), although there was a significant decrease in triglycerides of both groups and cholesterol in the control group before and after treatment. This result is in agreement with that in the study by El-Gharabawy et al. as no obvious change of biochemical indexes between the test groups, including fasting blood sugar, HbA1c, total cholesterol, low-density lipoproteins, high-density lipoproteins, or triglycerides, was observed [16]. According to Singh et al., when using metformin to treat psoriasis patients with metabolic disorders, there was a statistically significant difference in blood glucose levels, cholesterol, and triglycerides (0.002, 0.001, and <0.001, respectively) before and after treatment [24]. The dose of metformin may account for this difference as Singh et al. used a dose of 1000 mg/day. In the present study, patients only received a dose of metformin of 500 mg/day because of a lack of specific treatment guidelines, as well as precautions against hypoglycemia or adverse effects on the liver and kidneys when used with MTX. Additionally, because of the small sample size of the present study, it may be more difficult to detect differences between the two study groups. No patient in this study experienced hypoglycemia during the duration of the study, suggesting that metformin does not present any additional problems in the treatment of psoriasis. Lu et al., who conducted a 17-year metformin safety study with psoriatic patients, found that there was no difference in mortality, severe psoriasis, or hospitalization for psoriasis, with and without metformin [25]. Even with metformin at a dose of 1000 mg/day, no flare-ups or hospitalizations were observed [25].

## 5. Conclusion

Combination therapy using metformin and MTX has potential to substantially improve the PASI index of psoriasis patients with metabolic syndrome. Further in-depth studies with a larger sample size are necessary to evaluate the efficacy and safety of this combination.

## Figures and Tables

**Table 1:** Risk factors of metabolic syndrome.

Factors	Values
Waist circumference	≥90 cm in males and ≥80 cm in females
Triglycerides	≥150 mg/dl (1.7 mmol/L)
High-density lipoprotein cholesterol (HDL-C)	≤40 mg/dl (0.9 mmol/L) in males and ≤50 mg/dl (1.0 mmol/L) in females
Hypertension	Systolic pressure ≥130 mmHg or diastolic pressure ≥85 mmHg
Fasting glucose	≥100 mg/dl (5.55 mmol/L)

**Table 2:** Research group characteristics.

Indexes	Experiment group (*n* = 35)	Control group (*n* = 31)	*p*
Age	51.4 ± 13.2	50.5 ± 8.7	0.86
Disease duration	38.1 ± 10.1	38.5 ± 14.2	0.68
PASI	21.8 ± 8.3	22.3 ± 18.3	0.92

*Note.* Age, disease duration, and PASI of the patients in the two study groups are similar (*p* > 0.05, Student *t*-test).

**Table 3:** Treatment results of the study group (*n* = 35).

After (month)	PASI (before treatment) (*X* ± SD)	PASI (after treatment) (*X* ± SD)	% Reduction	*p*
1	21.8 ± 8.3	16.9 ± 7.7	22.48	0.01
2		14.3 ± 7.4	34.40	0.0002
3		9.0 ± 8.5	58.72	<0.001

*Note.* Mean PASI decreased each month. After three months of treatment, the mean PASI dropped by 58.72%. There was a statistically significant difference in PASI before and after treatment (*p* < 0.05, Student *t*-test).

**Table 4:** Treatment outcome of the control group (*n* = 31).

After (month)	PASI (before treatment (*X* ± SD)	PASI (after treatment) (*X* ± SD)	% Reduction	*p*
1	22.3 ± 18.3	18.1 ± 14.5	19.28	0.31
2		14.1 ± 10.9	36.78	0.03
3		11.1 ± 8.6	50.22	0.002

*Note.* Mean PASI decreased each month. After three months of treatment, the mean PASI dropped by 50.22%. There was a statistically significant difference in PASI before and after treatment (*p* < 0.05, Student *t*-test).

**Table 5:** Results of treatment in two groups according to the rate of PASI reduction after 3 months (*n* = 66).

Group	Result				
	Very good	Good	Moderate	Medium	Bad/No effect
Experiment (*n* = 35)	10 (28.6%)	2 (5.7%)	8 (22.9%)	10 (28.6%)	5 (14.2%)
Control (*n* = 31)	0 (0%)	0 (0%)	17 (54.9%)	13 (41.9%)	1 (3.2%)
*p*	<0.05				

*Note.* After 3 months of treatment, in the study group, 34.3% of the patients achieved “very good” and “good” results, whereas the control group had no patients with good results or more (*p* < 0.05, Chi square test).

**Table 6:** AST, ALT, and GGT before and after treatment of the two groups.

Index	Experiment group (*n* = 35)		*p*	Control group (*n* = 31)		*p*
	Before treatment	After treatment		Before treatment	After treatment	
AST	36.6 ± 21.8	32.02 ± 20.1	0.36	25.3 ± 10.7	26.4 ± 8.7	0.66
ALT	37.9 ± 30.2	39.1 ± 45.7	0.91	26.9 ± 17.3	30.9 ± 14.8	0.33
GGT	41.2 ± 36.6	34.6 ± 26.1	0.39	25.1 ± 15.7	40.2 ± 20.9	0.002

*Note.* There were differences in liver enzyme indexes (AST, ALT, and GGT) between two groups of patients before and after treatment. However, only the GGT index in the control group showed a significant difference (*p* < 0.05, Student *t*-test).

**Table 7:** Comparison of treatment results of two groups according to the venous blood glucose level.

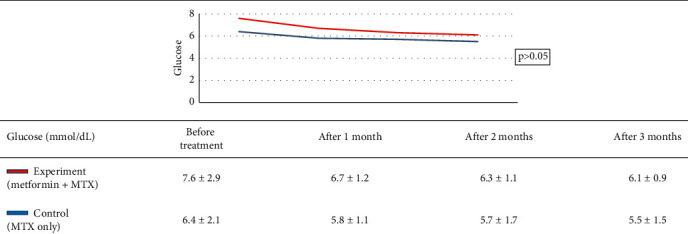

*Note.* After three months of treatment, the venous blood glucose levels of the study group and the control group markedly decreased (*p* < 0.05, Student *t*-test). However, the change in the venous blood glucose level between two groups was insignificantly different (*p* > 0.05, GEE regression).

**Table 8:** Comparison of treatment results of two groups according to the triglycerides index.

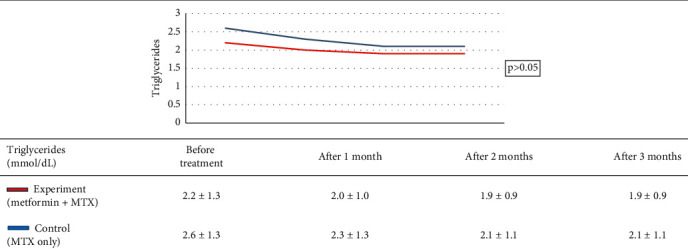

*Note.* After treatment, the triglycerides index of both groups dropped with a significant difference (*p* < 0.05, Student *t*-test). However, comparing the results of the two groups showed no statistical difference (*p* > 0.05, GEE regression).

**Table 9:** Comparison of treatment results of two groups according to the HDL cholesterol index.

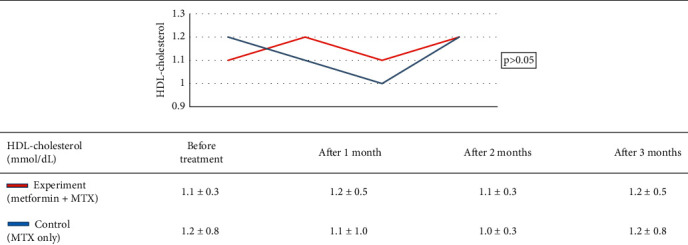

*Note.* After treatment, there was not a significant difference in the change of HDL cholesterol between the two groups (*p* > 0.05, GEE regression).

**Table 10:** Comparison of treatment results of two groups according to total cholesterol.

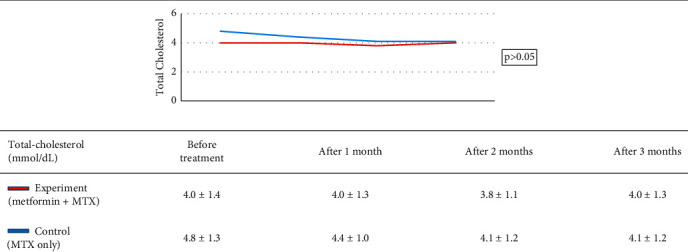

*Note.* After the treatment, there was a significant difference in cholesterol change between the experiment and control groups (*p* > 0.05, GEE regression). The control group total cholesterol index decreased (*p* < 0.05, Student *t*-test) while the intervention group remained unchanged (*p* > 0.05, Student *t*-test).

## Data Availability

Due to privacy and ethical concerns, neither the data nor the source of the data can be made available.
